# *Burkholderia pseudomallei* in soil and natural water bodies in rural Sri Lanka: A hidden threat to public health

**DOI:** 10.3389/fvets.2022.1045088

**Published:** 2023-01-17

**Authors:** Himali S. Jayasinghearachchi, Thilini A. Muthugama, Jayanthi Masakorala, Upeksha S. Kulasekara, Kumari Jayaratne, D. A. Dasun N. Jayatunga, Aruna D. De Silva, Enoka M. Corea

**Affiliations:** ^1^Institute for Combinatorial Advanced Research and Education, General Sir John Kotelawala Defense University, Dehiwala-Mount Lavinia, Sri Lanka; ^2^Biomedical Laboratory 2, Faculty of Medicine, General Sir John Kotelawala Defense University, Dehiwala-Mount Lavinia, Sri Lanka; ^3^Department of Medical Microbiology and Immunology, Faculty of Medicine, University of Colombo, Colombo, Sri Lanka

**Keywords:** melioidosis, *Burkholderia pseudomallei*, Sri Lanka, natural water, environment

## Abstract

*Burkholderia pseudomallei* is the causative agent of the potentially fatal infection, melioidosis. This study provides the first evidence for the presence of *B. pseudomallei* in soil and water in Sri Lanka. Targeted sampling of soil and natural water sources was done between November 2019 and October 2020 over eight field visits encompassing the neighborhood of 28 culture and/or antibody-positive melioidosis patients in northwestern, western and southern Sri Lanka. A total of eight environmental isolates of *B. pseudomallei* (BPs-env1 to BPs-env8) were cultured from 116 soil and 117 natural water samples collected from 72 locations. The presence of *B. pseudomallei* in soil and natural water in these areas poses a risk of melioidosis for populations cultivating crops in such soils and using untreated water from these sources for drinking, bathing, and other domestic purposes. Identifying sites positive for *B. pseudomallei* may help to mitigate risk by raising public awareness of contaminated environmental sources and allowing soil and water remediation.

## Introduction

Melioidosis, a potentially fatal infection of humans and animals, is a tropical disease with a non-specific presentation, ranging from overwhelming sepsis to community acquired pneumonia to chronic localized infection. Multifocal involvement of more than one system and abscess formation, especially in the liver, spleen, and prostate, are common (1). *Burkholderia pseudomallei*, the causative agent of melioidosis, is an aerobic, non-spore-forming, non-fermenting, Gram-negative environmental bacterium widely distributed in the rhizosphere, soil and water (1). Infection is acquired *via* exposure to soil or water by inoculation, inhalation, or ingestion (2). Agricultural workers and rural populations in endemic areas are at high risk (3).

A number of studies have highlighted the potential hazards associated with the presence of *B. pseudomallei* in natural water bodies and its ability to survive and proliferate in low nutrient environments such as unchlorinated drinking water sources (4, 5) and the bacterium has been detected in environments adjacent to culture-positive melioidosis patients (6–8). Inglis and Sousa have drawn attention to the public health implications of melioidosis and recommended targeted environmental threat assessment (9).

Many studies have shown that *B. pseudomallei* is phylogenetically diverse. Multilocus sequence typing (MLST) is a useful epidemiological tool to investigate the biogeographic and phylogenetic features of *B*. *pseudomallei* and has been used widely to identify the population genetics of isolates from a particular geographic area and to analyze their relationship to isolates from other regions (1). Sequence type (ST) data can be further analyzed using phylogenetic tools such as PHYLOVIZ to elucidate the genetic relatedness of *B. pseudomallei* from distinct locations (10). MLST has also been used to identify the source of outbreaks although the restricted number of genes analyzed may limit discrimination as demonstrated by whole genome sequencing (WGS) of identical STs revealing non-clonality in a case cluster (11). MLST and WGS have been employed to explore the genetic relationship between clinical and environmental isolates of *B. pseudomallei* in Australia (12, 13). A correlation between ST and virulence has not been found to date.

Variably present molecular markers such as the *Burkholderia thailandensis*-like flagellum and chemotaxis gene cluster (BTFC) and *Yersinia*-like fimbrial (YLF) gene cluster have been used to characterize *B. pseudomallei* populations in different endemic regions. For example, Australian *B. pseudomallei* isolates are more likely to encode the ancestral BTFC gene cluster whereas isolates from Asia almost exclusively carry the YLF gene cluster (14). In addition, variably present virulence factors such as the actin-based motility gene, *bimA*, lipopolysaccharide (LPS) O antigen, and filamentous hemagglutinin gene (*fhaB*) have been found to be useful in molecular characterization of *B. pseudomallei* in different geographical regions. The *bimA* gene is found in two different allele variants (typical *B. pseudomallei*-like *bimA*_Bp_ and atypical *B. mallei*-like *bimA*_Bm_) and a differential distribution of these two variants in endemic regions has been reported. In the Australian population, 12% of *B. pseudomallei* isolates contain *bimA*_Bm_ whereas in Thailand *bimA*_Bm_ has not been reported. The *fhaB* allele has been found in three different forms. All Thai strains contain the *fhaB3* gene while only 83% of Australia strains are positive (15). LPS O-antigen of *B. pseudomallei* is highly diverse and grouped by serotype into types A, B, and B2 (16).

Melioidosis is endemic in Sri Lanka with a wide geographic and demographic distribution. A large number of *B. pseudomallei* clinical strains have been isolated from melioidosis patients throughout the country (3). Sequence typing of 193 *B. pseudomallei* clinical isolates from Sri Lanka revealed 71 distinct STs with ST1137, ST1136 and ST1132 being the most frequent. In addition, examination of variably present genetic markers i.e., *bimA, fhaB3*, YLF, and BTFC gene clusters and LPS type A among 310 isolates showed a diversity of genotypes, intermediate between Southeast Asia and Oceania (10).

Successful culture isolation of *B. pseudomallei* from soil and water is fraught with difficulties including the presence of the bacterium in the environment in a viable but non-culturable state and overgrowth by other soil bacteria, even with the use of selective media. Other factors influencing culture isolation include the depth of soil sampling, timing of sampling and volume of sample (1). A consensus guideline for soil sampling has been proposed (17) but requires a soil sampling depth of 30 cm that is both time and labor intensive. There is no guideline for isolation of *B. pseudomallei* from water but bacterial concentration by filtration has been used successfully (2).

In 2015, a cluster of melioidosis cases caused by genetically diverse strains was reported after heavy rainfall in the eastern part of Sri Lanka (11). A similar cluster followed strong monsoonal winds in 2017 (18). The occurrence of these case clusters indicates the presence of *B. pseudomallei* in the environment. However, the bacterium has never been isolated from soil or water in Sri Lanka. Knowledge on the distribution of *B. pseudomallei* in natural environments and its environmental persistence is of importance to understand the epidemiology of melioidosis and identification of *B. pseudomallei* contaminated environments is essential to determine potential risk areas, identify occupational groups that are frequently exposed to such environments and mitigate risk from such exposures.

This study reports the first isolation of *B. pseudomallei* from soil and natural water in the western, northwestern and southern parts of Sri Lanka employing targeted sampling of potential environment sources around the homes and workplaces of melioidosis patients and examines the genetic relatedness of the environmental isolates to the corresponding clinical isolates and to environmental isolates from other endemic regions.

## Methods

### Collection and processing of soil and natural water samples

Targeted sampling of soil and natural water suspected to be the source of infection was done in the course of eight field visits to 28 culture and/or antibody-positive melioidosis patients between November 2019 and October 2020. The patients (or relatives in the case of deceased individuals) were interviewed to identify the suspected source of exposure and appropriate soil and water specimens were obtained. If the household well or natural spring was identified as the likely source, ~250 mL of water was collected into pre-labeled sterilized polypropylene bottles. If the source was a natural water ditch, the water sample was collected using a hand spade. Soil sampling was done from the surface at depths varying from 0 to 30 cm, with ~100 g of soil from each sampling point collected into sterile, pre-labeled, polypropylene bags while one teaspoonful of soil was inoculated into 10 mL of modified Ashdown's broth *in situ* (19). Sample collection utensils were washed with bottled water, disinfected with 70% ethanol and dried between samples. Soil and water samples were transported to the laboratory on the same day under ambient conditions, protected from direct sunlight. Global positioning system (GPS) coordinates of each sampling location were recorded using GPS Tracker and mapped on Google Earth using ArcGIS 10.2.

Soil enrichment broth cultures were incubated at 37°C for 72 h and a loopful of broth, from the surface was inoculated on Ashdown's selective agar (20). Water samples were filtered through a 0.45 μm nitrocellulose membrane filter using a Millipore Filtration Unit and the filter membrane was aseptically transferred into a sterile 50 ml conical tube containing 10 ml of modified Ashdown's broth using a sterile forceps and incubated at 37°C in ambient air for 3–4 days. A loopful of broth, taken from the surface, was spread on Ashdown's selective agar and incubated at 37°C in ambient air for 3–4 days. *B. pseudomallei* colonies were identified based on their characteristic colony morphology and presumptive colonies were sub-cultured on fresh plates. Cultures were confirmed using a real-time polymerase chain reaction (RT-PCR) *lpx*O assay using DNA extracted by heat lysis (21) with DNA from a *B. pseudomallei* clinical isolate previously confirmed by whole genome sequencing as the positive control (22). Isolates were further confirmed by latex agglutination (23).

### MLST and PHYLOVIZ analysis

Amplification of seven housekeeping genes (*ace-gltB-gmhD-lepA-lipA-narK-ndh*) was performed using established oligonucleotide primer sequences as reported previously (24). PCR products were submitted for amplicon purification and SANGER sequencing to Macrogen Inc., Seoul, South Korea. Each DNA fragment was sequenced in forward and reverse directions using the same oligonucleotide primers that were used for the initial PCR amplification. The batch sequence (7 loci) from each isolate was queried in the MLST database (http://pubmlst.org/bpseudomallei.mlst.net/) to determine the allelic profile. The allelic profile of each isolate was queried for a match to the existing STs on the MLST database. PHYLOVIZ 2.0 available at PubMLST (http://pubmlst.org/bpseudomallei.mlst.net/) was used to determine the genetic relationship of STs of Sri Lankan environmental isolates of *B. pseudomallei* in relation to environmental STs (*n* = 454) from Australia (*n* = 220), Thailand (*n* = 163), Vietnam (*n* = 25), Laos (*n* = 19), China (*n* = 11), Cambodia (*n* = 8), Singapore (*n* = 4), India (*n* = 2), Taiwan (*n* = 1), and Bangladesh (*n* = 1).

### Genotyping of *B. pseudomallei*

Genotyping to detect the variably present gene markers YLF) and BTFC, *fhaB*3, *bimA* gene variants *bimA*_BP_/*bimA*_BM_ and LPS O-antigen type A (LPSA) was done using RT-PCR assays with gene-specific oligonucleotide primers (14–16). BRYT green qRT-PCR master mixture (Promega, USA) was used in this study. Previously confirmed *B. pseudomallei* isolates were used as positive controls (22).

## Results

### Isolation of *B. pseudomallei* from soil and natural water

**Figure 1:**
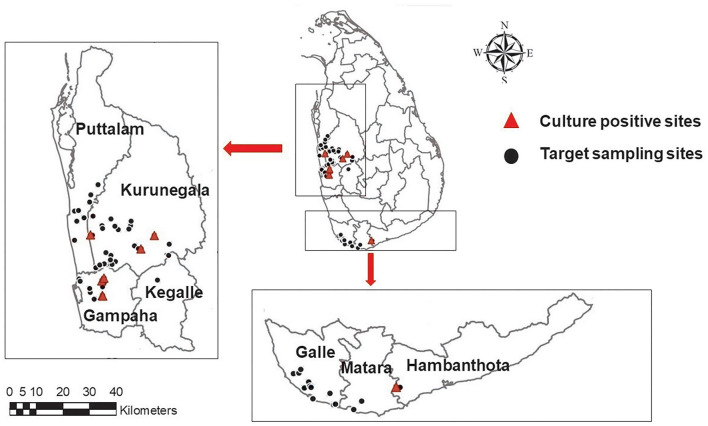
A map showing the geographical distribution of targeted sampling sites in western, northwestern, and southern parts of Sri Lanka; (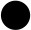)-target sampling sites, (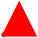) culture positive sites. Maps of the study region were generated using ArcGIS software version 10.3.1.

**Figure 2:**
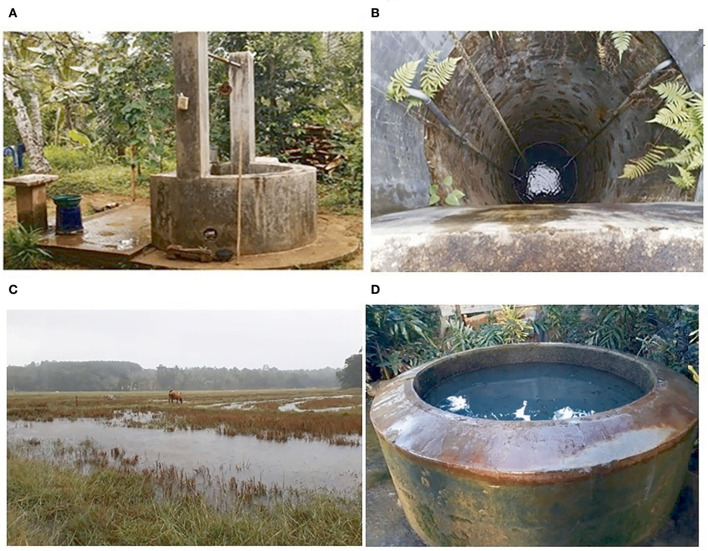
Natural water sources of *Burkholderia pseudomallei*. **(A, B)** unchlorinated domestic well **(C)** Stagnant water in paddy field **(D)** perennial spring “Bubula”.

A total of eight environmental isolates of *B. pseudomallei* (BPs-env1to BPs-env8) were cultured from 116 soil and 117 natural water samples collected from 72 sites in northwestern, western, and southern Sri Lanka (Figure 1). Six isolates were from water, giving an isolation rate of 6/116 (5%), with three samples being from unchlorinated domestic wells and one each from still water in a paddy field, stagnant water from a garden and a perennial spring (Figure 2). Both soil isolates (isolation rate of 2/117, 1.7%) were from paddy fields. All the isolates were obtained from the environs of seven patients, six of whom survived (Table 1).

**Table 1:** Description of genotypes of environmental and corresponding clinical isolates, isolation source, geographic location, and clinical presentations.

**Environmental isolate and related clinical isolate**	**ST**	**YLF/BTFC**	***BimA*bm/*bimA*Bp**	**LPSA**	***fhaB3***	**Isolation source**	**Geographic location of melioidosis patient (Province) [GPS coordinates]**	**Clinical features and (clinical outcome)**
BPs-env1	1435	YLF	*bimA*_Bp_	+	–	Unchlorinated domestic well water	Pahala Madagoda (NWP)	Liver abscess (Recovered)
BPs351	1132	YLF	*bimA*_Bp_	+	–	Pus from liver abscess	[7.41359, 80.20755]	
BPs-env2	1137	YLF	*bimA*_Bp_	–	–	Soil from paddy field	Ihala Madawala (NWP)	Wound infection, cellulitis, sepsis (Died)
BPs527	1132	YLF	*bimA*_Bp_	–	–	Blood	[7.4883911, 80.282942]	
BPs-env3 antibody positive only. No culture isolated	1,132	YLF	*bimA*_Bp_	+	–	Soil from paddy field	Dummalasuriya (NWP) [7.4920723, 79.9183956]	Sepsis (Recovered)
BPs-env4	1435	YLF	*bimA*_Bp_	+	+	Unchlorinated domestic well water	Dunagaha Palliyapitiya (WP)	Axillary abscess (Recovered)
BPs549	1139	BTFC	*bimA*_Bp_	+	–	Pus from axillary abscess	[7.2289897, 79.983436]	
BPs-env5	1435	YLF	*bimA*_Bp_	+	–	Unchlorinated domestic well	Hunumulla (WP)	Parotid abscess (Recovered)
BPs531	1478	YLF	*bimA*_Bp_	+	–	Pus from parotid abscess	[7.245659, 79.99631]	
BPs-env6	1137	YLF	*bimA*_Bp_	+	–	Stagnant water in garden	Goigama (WP)	Sepsis and pneumonia (Recovered)
BPs-env7	1137	YLF	*bimA*_Bp_	+	–	Water from paddy field	[7.1478937, 79.9871442]	
BPs547	1137	YLF	*bimA*_Bp_	+	–	Blood	[7.145316, 79.9886379]	
BPs-env8	1137	YLF	*bimA*_Bp_	+	+	Perennial spring water	Kumbalgoda (SP)	Sepsis and lung abscess (Recovered)
BPs564	1137	YLF	*bimA*_Bp_	+	+	Blood	[6.0738946, 80.6803721]	

BPs, clinical isolates; BPs, env-environment isolates; YLF, Yersinia-like fimbrial; BTFC, B. thailandensis-like flagellum and chemotaxis; bimA_BP_/bimA_BM_, Burkholderia intracellular motility A (bimA) gene variants; fhaB3, filamentous hemagglutinin 3; LPS type A, lipopolysaccharide O-antigen type A; NWP, North Western Province; WP, Western Province; SP, Southern Province.

### Genetic relatedness of the environmental isolates to the corresponding clinical isolates

Four isolates belonged to ST1137, three to ST1435 and one to ST1132 (Table 1). In the case of BPs-env1, BPs-env4, and BPs-env5 isolated from unchlorinated domestic drinking water wells and BPs-env2 from paddy soil, the sequence type (ST) of the environmental isolate differed from the corresponding clinical isolate. However, BPs-env6 and BPs-env7, isolated from stagnant garden water and paddy field water of a single patient, shared the same ST (ST1137) and possessed identical genotypes (YLF, *bimA*_BP_, LPSA positive, and *fhaB3* negative) with the related clinical isolate. BPs-env8 was isolated from a perennial spring, “Bubula.” Villagers use water from this spring for washing agricultural utensils and machinery. The corresponding melioidosis patient was residing approximately 150 m away and used water from the “Bubula” spring to wash his motorcycle. In this case, too, the clinical isolate was identical in terms of ST (1137) and genotypes (YLF, *bimA*_BP_, LPSA positive, and *fhaB*3 positive) with the environmental isolate from the spring although soil and water samples obtained from his residence and domestic well were negative for *B. pseudomallei*.

### Genetic relatedness of the environmental isolates to environmental isolates from other endemic regions

**Figure 3:**
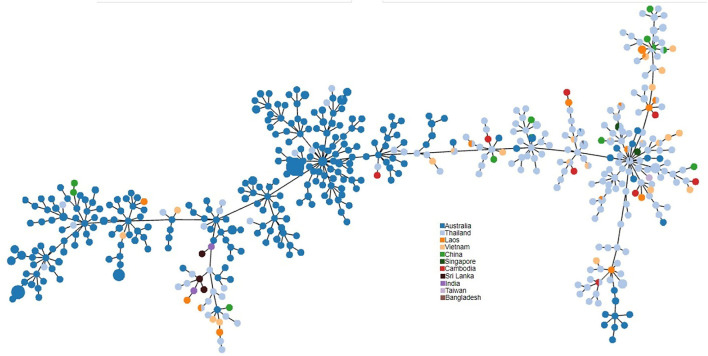
PHYLOViZ analysis of STs showing the phylogenetic relationship of Sri Lankan environment isolates (*n* = 3 STs) with environment isolates from Australia (*n* = 220), Thailand (*n* = 163), Vietnam (*n* = 25), Laos (*n* = 19), China (*n* = 11), Cambodia (*n* = 8), Singapore (*n* = 4), India (*n* = 2), Taiwan (*n* = 1), and Bangladesh (*n* = 1).

PHYLOViZ analysis of the phylogenetic relationship of Sri Lankan environmental isolates with other selected environmental isolates revealed two distinct clusters i.e., South Asian strains and Australian strains. Sri Lankan environmental isolates clustered with Australian environmental isolates (Figure 3).

## Discussion

Melioidosis may be acquired by direct contact with contaminated soil, ingestion of untreated water or inhalation of dust or contaminated aerosols. Individuals with underlying diseases such as diabetes, renal, or liver disease and various forms of immunosuppression are most at risk (1). The bacterium is commonly found in the soil and stagnant water of paddy fields and wetlands in endemic regions and contaminated soil and water has been shown to be the most important source of infection (12). An association between melioidosis and paddy cultivation and the presence of *B. pseudomallei* in drinking water has been shown (2).

The bacterium has been isolated from a wide range of environment niches including natural water environments and unchlorinated drinking water wells in endemic regions. The unusual ability of *B. pseudomallei* to thrive in harsh environments has been shown to contribute to its long-term persistence in such environments (25).

This study reports the first evidence for the presence of *B. pseudomallei* in soil and natural water in Sri Lanka. Notably, it demonstrates that taking a careful history of exposure from the patient can help to pinpoint sites where the bacterium is present.

The presence of *B. pseudomallei* in natural water sources in Sri Lanka poses a major public health issue as the proportion of households with access to pipe-borne treated water is currently below 50% (www.waterboard.lk). Water from household wells is commonly used for drinking, cooking and bathing, and the high proportion of melioidosis cases presenting with parotid and abdominal abscesses in Sri Lanka may be a reflection of transmission by ingestion of contaminated water (3).

In addition to dug wells, natural perennial bubbling springs, such as the “Bubula” sampled during this study, are often enclosed and used for community needs. Water from such springs continuously overflows into the surrounding environment, posing a high risk of contamination and spread of the pathogen to distant areas during flooding and *via* strong winds (26, 27).

MLST of the eight environmental isolates revealed only three STs, showing a low diversity among the environmental strains as has been noted in Singapore (1). There were no novel STs, when compared with the international *B. pseudomallei* database (https://pubmlst.org/organisms/burkholderia-pseudomallei) and all three sequence types had been recorded previously in clinical infections in Sri Lankan patients (10). PHYLOViZ analysis of selected environmental STs from the *B. pseudomallei* database divided them into two distinct populations, Australian and Southeast Asian. The Sri Lankan environmental isolates clustered with the Australian isolates. It is interesting to note that the Australian population is recognized as the most ancestral population (28). The reason for the close relationship between *B. pseudomallei* isolates from Sri Lanka and Australia is unknown but could be related to their original positions in Gondwanaland (10).

BPs-env6 and BPs-env7, isolated from the patient's immediate environs (stagnant water in the garden and paddy water, respectively), and BPs-env8 recovered from a community spring shared ST 1137 and genotypes with the clinical isolates from the corresponding patients which may indicate direct transmission of the pathogen from the environment (29–31). However, since 1137 is a widespread ST in Sri Lanka, whole genome sequencing will be required to confirm that the clinical and environmental isolates are, indeed, clonal in nature. The presence of non-identical genetic patterns among the clinical and environmental isolates in the other four cases, suggests significant environmental contamination with multiple strains, as reported previously (32).

The almost ubiquitous presence of *B. pseudomallei* in the environment of endemic countries and universal exposure of resident agricultural populations pose a challenge to the prevention and control of melioidosis. On a local scale, if contamination of water sources is confirmed by culture, preventive measures, such as chlorination of wells or filtering or boiling water before drinking can be recommended to minimize the risk of acquiring melioidosis (2).

## Conclusion

This study confirms the presence of *B. pseudomallei* in soil and natural water in Sri Lanka, placing the populations using untreated water from such sources at risk of melioidosis. Although no vaccine is currently available, advising the public of the dangers associated with consumption of untreated water and raising awareness among clinicians that melioidosis should be considered in the differential diagnosis of febrile illness in areas without a pipe-borne, treated water supply may help to reduce transmission and enhance early diagnosis. Identifying specific environmental sites positive for *B. pseudomallei* may assist to mitigate risk *via* soil and water remediation to reduce the bacterial burden.

## Data availability statement

The original contributions presented in the study are included in the article/supplementary material, further inquiries can be directed to the corresponding authors.

## Ethics statement

Ethics approval for this study was obtained from the Ethics Review Committee, Faculty of Medicine, University of Colombo, Sri Lanka (EC-17-020).

## Author contributions

HJ and EC contributed to the conceptualization and design of the study. TM, UK, KJ, JM, and DJ collected and organized the data. TM and HJ analyzed the data. HJ wrote the original draft. EC, HJ, and DJ contributed to the interpretation of the results and manuscript revision. All authors contributed to the article and approved the submitted version.
